# Supplementary material for: Place cells are more strongly tied to landmarks in deep than in superficial CA1
Source: Nat Commun. 2017 Feb 20;8:14531. doi: 10.1038/ncomms14531 (PMC5321734; doi:10.1038/ncomms14531)
Supplement: Supplementary Information — Supplementary Figures [file ncomms14531-s1.pdf]

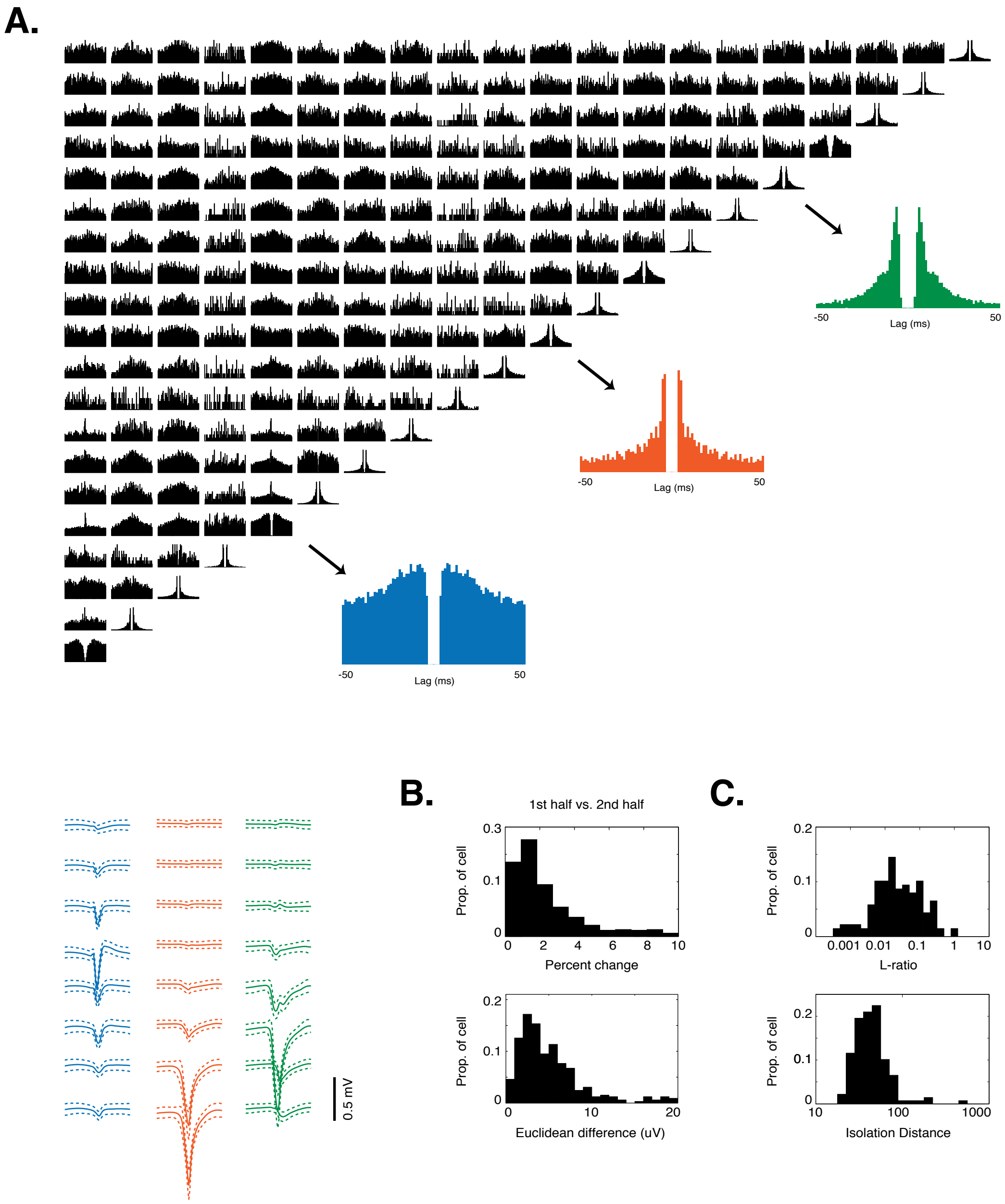

Supplementary Figure 1

Spike sorting and isolation quality of clusters.

A: (Top); Example of auto-correlograms and cross-correlograms of 20 CA1 units recorded simultaneously. (Bottom); Average (± s.d.) of unit waveforms. **B**: Comparison between the average spike amplitudes of the first and second halves of all the recording sessions. (Top); Percent change on the channel with the highest amplitude. (Bottom); Change in the Euclidean distance between the amplitudes on all the channel. **C**: Distribution of L-ratio measures and Isolation Distance index.

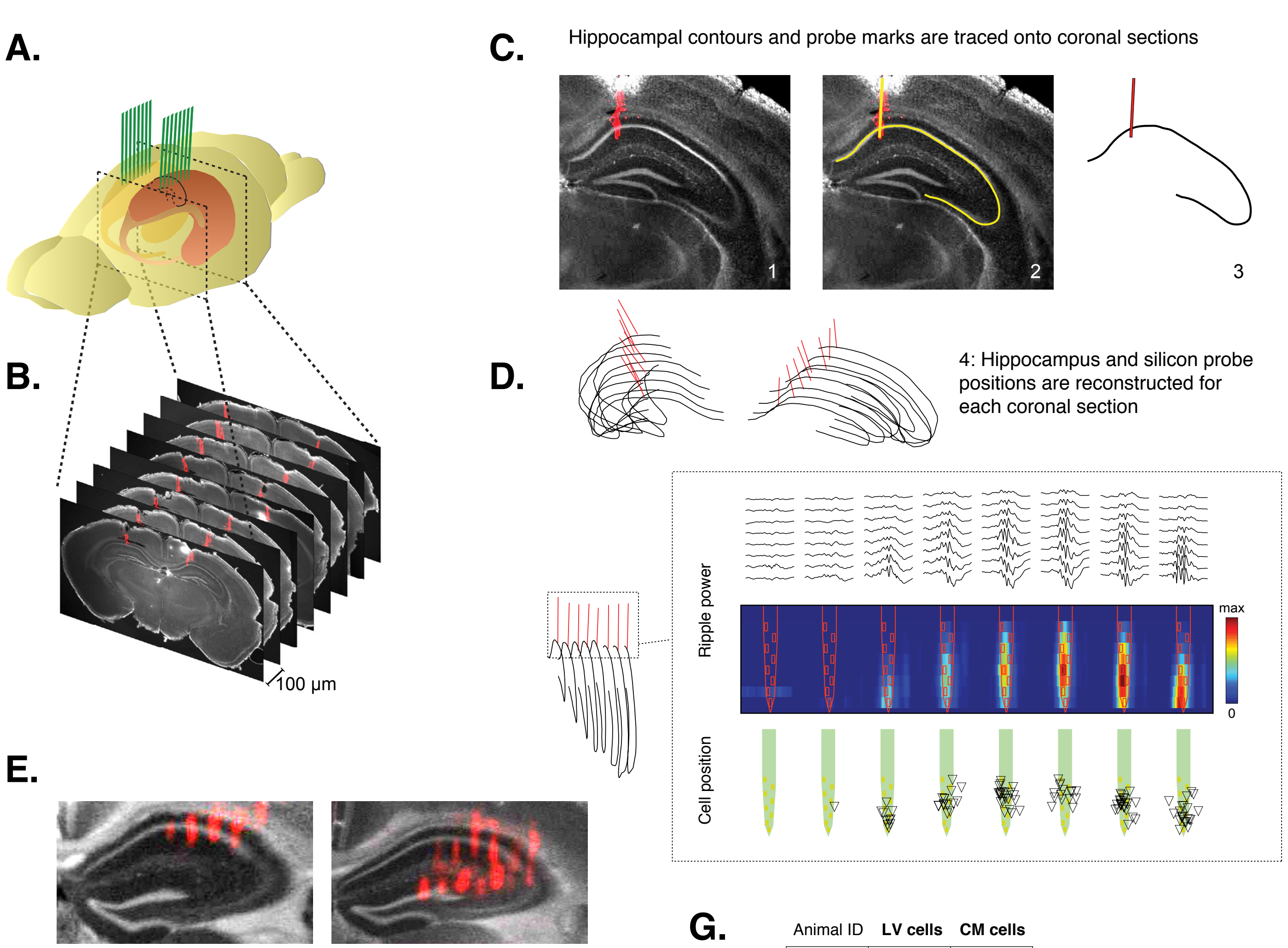

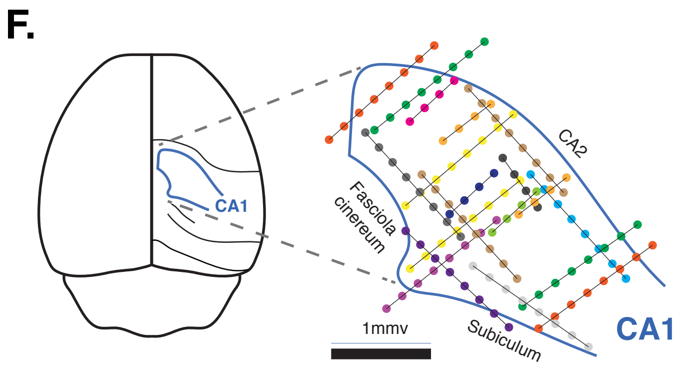

G.

	Animal ID	LV cells	CM cells
CA1	TG08	7	20
	TG10	9	23
	TG11	11	33
	TG15	22	35
	TG16	9	22
	TG18	6	19
	TG23	34	31
	TG24	31	35
	TG25	26	29
	TG26	1	3
	TG39	7	9
	TG40	17	13
	TG41	4	4
	TG42	10	7
	TG43	3	4
	TG44	12	12
CA3	TG08	0	9
	TG10	0	6
	TG11	1	4
	TG16	1	19
	TG18	1	27
	TG22	0	5

Supplementary Figure 2

3D anatomy, electrode track reconstruction and cell location

A: Scheme of the mouse brain showing the hippocampus and position of the shanks of silicon probes. **B**: Example of coronal sections with the position of the silicon probes' shanks in red. Image reconstructed by overlaying DAPI and DiI fluorescence images. **C**: CA contour on each slice were drawn using customized Matlab routine (1-2-3). Traces were scaled, aligned and visualized in 3D (4). **D**: Example of CA1 ripple activity (top), ripple power (middle) and putative position of recorded cells (bottom) (See Methods). **E**: Example of coronal sections with silicon probes shanks oriented along the proximodistal axis, targeting CA1 (top) and CA3 (bottom). **F**: Summary diagram of shanks' locations for all experiments. Dots connected by lines correspond to the shanks from the same silicon probe. **G**: Number of LV cells and CM cells recorded in CA1 and CA3 for different mice.The color code correponds to the ones in **F**.

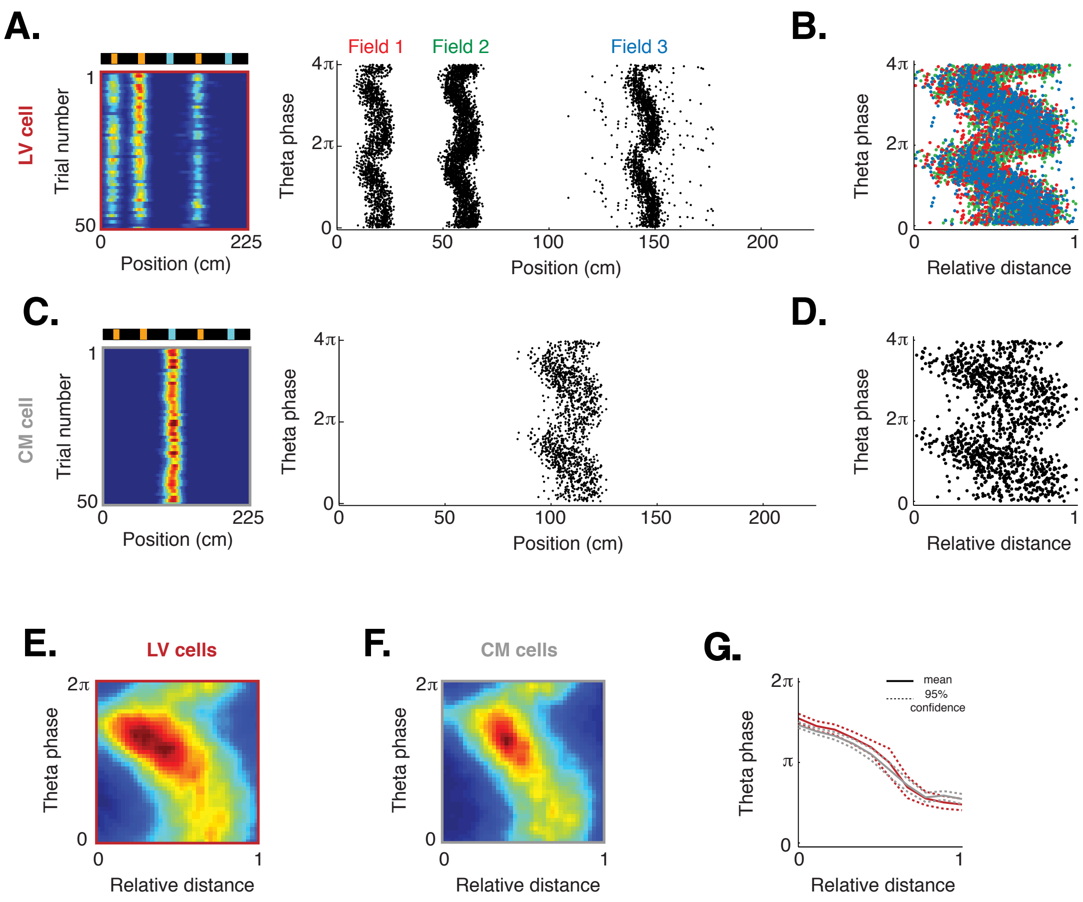

Supplementary Figure 3

Theta phase precession in Landmark-vector cells and Context-modulated cells

A: Example of theta phase precession in LV cell. Position on the belt is plotted on the x axis, and the theta phase at which each spike occured is plotted on the y axis. **B**: Overlay of normalized position within each field. **C**,**D**: Same as **A**, **B**, for CM cells. **E**: Theta phase precession depicted for all the spikes of LV cells. **F**: Same as **E** for CM cells. **G**: Circular mean and confidence interval for all the fields.

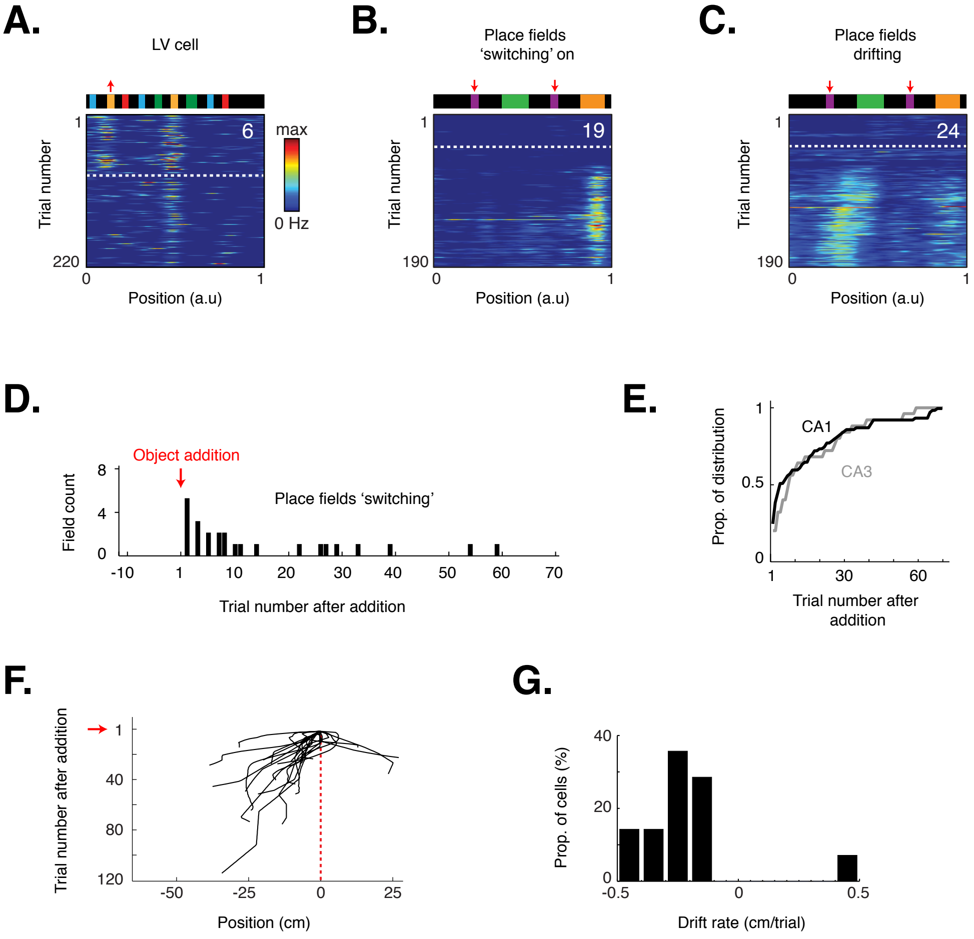

H.

	Animal ID	**# cells drifting**	**#cells switching**
**CA1**	TG08	4	13
	TG10	13	0
	TG11	7	23
	TG15	14	21
	TG16	4	15
	TG18	5	8
**CA3**	TG08	3	4
	TG16	7	7
	TG18	10	12
	TG22	1	2

Supplementary Figure 4

Cell activity and dynamics in CA3

A,B,C: Example of LV cell **(A)**, switching **(B)** and drifting **(C)** cells in CA3. **D**: Field emergence of switching cells as a function of trials. **E**: Cumulative distribution of **(D)** for CA1 and CA3 (P=0.81, unpaired Kolmogorov-Smirnov test). **F**: Trajectories of drifting place fields along the trials. Field drift starting positions are aligned on 0. **G**: Distribution of drift rates. **H**: Number of drifting and switching cells, in CA1 and CA3, for different mice.

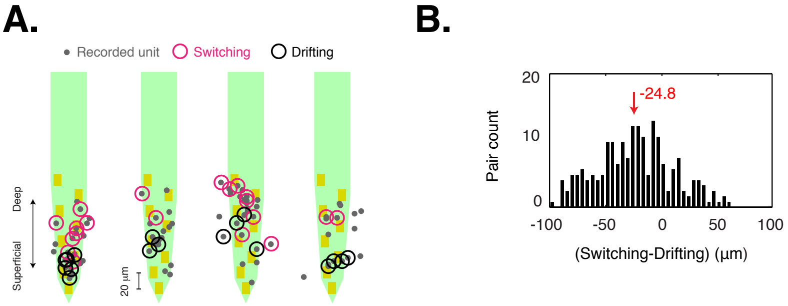

Supplementary Figure 5

Relative repartion of switching and drifting cells along CA1 radial axis

A: Examples of shanks from four different silicon probes with cell positions. **B**: Distribution of depth-differences between pairs of neurons from the same shank. Red arrow indicates the mean. (Mean of the difference versus 0, P<0.001, one-tailed t-test)
